# Novel Application of Cyclolipopeptide Amphisin: Feasibility Study as Additive to Remediate Polycyclic Aromatic Hydrocarbon (PAH) Contaminated Sediments

**DOI:** 10.3390/ijms12031787

**Published:** 2011-03-09

**Authors:** Anne Groboillot, Florence Portet-Koltalo, Franck Le Derf, Marc J. G. Feuilloley, Nicole Orange, Cécile Duclairoir Poc

**Affiliations:** 1 Laboratory of Cold Microbiology-Signals and Microenvironment, University of Rouen, EA 4312, 55 rue Saint Germain, 27000 Evreux, France; E-Mails: anne.groboillot@univ-rouen.fr (A.G.); marc.feuilloley@univ-rouen.fr (M.J.G.F.); nicole.orange@univ-rouen.fr (N.O.); 2 UMR 6014 COBRA, University of Rouen, 55 rue Saint Germain, 27000 Evreux, France; E-Mails: florence.koltalo@univ-rouen.fr (F.P.-K.); franck.lederf@univ-rouen.fr (F.L.D.)

**Keywords:** biosurfactant, solubility enhancement, polycyclic aromatic hydrocarbons, bioremediation, dredged sediments

## Abstract

To decontaminate dredged harbor sediments by bioremediation or electromigration processes, adding biosurfactants could enhance the bioavailability or mobility of contaminants in an aqueous phase. Pure amphisin from *Pseudomonas fluorescens* DSS73 displays increased effectiveness in releasing polycyclic aromatic hydrocarbons (PAHs) strongly adsorbed to sediments when compared to a synthetic anionic surfactant. Amphisin production by the bacteria in the natural environment was also considered. DSS73’s growth is weakened by three model PAHs above saturation, but amphisin is still produced. Estuarine water feeding the dredged material disposal site of a Norman harbor (France) allows both *P. fluorescens* DSS73 growth and amphisin production.

## 1. Introduction

Recently, increasing interest has been given to biosurfactants, as these metabolites are produced by a wide variety of microorganisms [1–8]. Their amphiphilic structure leads them to accumulate at the interfaces, thus they are able to increase solubility and diffusion of insoluble compounds in water. Compared to their synthetic counterparts, biosurfactants are known for their biodegradability, their reduced toxicity, and their environmental “friendliness” [9]. Moreover, their efficiency is often higher than conventional surfactants: a similar surface tension reduction is obtained by a smaller biosurfactant quantity [3]. Their efficiency has been also proven in extreme conditions (temperature, ionic strength, pH) [10–12].

Such properties allow the biosurfactants to be potential candidates in many industrial applications, especially in the food, cosmetics, agricultural chemicals, biomedical materials or in health care and cleaning industries, *etc*. They have also been tested in environmental applications [3,7,8,13,14]. Indeed more and more environmental regulations favor these bioproducts compared to synthetic surface-active compounds [15].

Essentially, biosurfactants are classified either as low or high-molecular-mass. Whereas high-molecular-mass biosurfactants consist in particulate and polymeric amphiphiles, the low molecular-mass biosurfactants deal with three major types:

- glycolipids or lipopolysaccharides: rhamnolipids, trehalolopids [16], sophorolipids [17,18];
- lipoproteins-lipopeptides: acyclic [19] and cyclic ones (cyclolipopeptides) [20,21];
- and hydroxylated crosslinked fatty acids (mycolic acids) or phospholipids [3].

Among them, rhamnolipids, produced by *Pseudomonas aeruginosa*, are intensively described in literature and their applications also [3,14,16,22,23]. However, our interest was retained by cyclolipopeptides (thereafter mentionned as CLPs), because of the variety of microorganisms, which produce them, mainly *Bacillus* and *Pseudomonas* bacteria [4,7,21,24] and their abundant variability of structures [21,24]. They are composed of several amino-acids linked to a molecule of hydroxyalkyl acid [25]. The first discovered was surfactin [26–29]. CLPs are especially attractive for industrial applications as they present good surface tension reduction with low critical micellar concentrations (CMC, need of minimum CLPs quantity to be efficient) and versatile bioactive properties. Thus, CLPs attract attention of food and biomedical industries [30,31].

Concerning environmental remediation of any organic or inorganic contaminants [14,32], biosurfactants present a great potential in oil or metal recovery, increasing bioavailability of low solubility compounds [7,19,33,34]. Most of the more persistent organic compounds, such as polycyclic aromatic hydrocarbons (PAHs) or polychlorinated biphenyls (PCBs), are toxic and carcinogenic [35]. Among all the PAHs, 16 individual PAH compounds, consisting of two to six fused aromatic rings, have been classified as priority pollutants by the United States Environmental Protection Agency [36] due to their chronic toxic effects. Various environmental matrices, such as air, water, vegetation, soil and sediments act as environmental sinks for PAHs. Indeed, high concentrations of PAHs in benthic and aquatic organisms, but also in sediments, have already been reported [37,38].

In the case of river or harbor sediment contamination, dredging seems to be the beginning of a solution for pollution remediation [39]. In France, materials dredged annually in the harbors and the channels represent about 50 × 10^6^ m^3^, of that 24 × 10^6^ m^3^ concentrate most of organic and inorganic contamination. Regulations have become more strict in Europe in the last decade. To protect the marine environment against ecological risks of dredging and capping, contaminated sludge can no longer be dumped at sea. The alternative is to store it in confinement sites, which are currently reaching capacity in Europe. Different options are now being explored in order to eliminate, or at least to weaken, the present contamination in sediments, to valorize them thereafter as filling or building materials, *etc*. The pretreatment processes primarily consist in a granulometric separation of the coarse fractions from the fine particles, where pollution is concentrated. After pretreatment, polluted fine slurries may be stored in confinement sites or submitted to processes elaborated for polluted grounds. The offered remediation strategies are mainly classified in four types: chemical, physical, or physical-chemical together, and biological [40]. Briefly, chemical treatments are largely based on oxidation with various oxidants. The physical-chemical processes are mainly extraction and washing. Washing treatments are less aggressive than physical treatments (e.g., incineration, thermal desorption, *etc.*) and thus less disturbing for the ground [41]. An alternative technique would also be an electrokinetic remediation (EK), which presents less important energy cost and is particularly adapted for fine sediments. A weak electric current, applied via electrodes inserted horizontally or vertically into the matrix to be treated, induces the mobility of the ionized species in the interstitial liquid phase towards the two electrodes permitting to reduce the contaminants level inside the sediments [42,43].

Finally, several bioremediation approaches are also proposed: first of all to diminish the risk associated to the remediation processes to human health, secondly to minimize the costs, and thirdly to be less invasive for the environment [44]. Bioremediation consists in soil detoxification by using either micro-organisms or biomass to biodegrade or absorb the pollutants. The principal processes are generally biostimulation, bioattenuation or bioaugmentation [45]. An extended definition of bioremediation would be soil or sediment amendment with additives from biological origins, (e.g., biosurfactant which contribute to the decontamination process).

To achieve better PAH removal efficiencies (extraction and/or degradation) and to pass through limitations of individual remediation techniques, physical, chemical and biological treatments can be combined [40]. For instance, the combination of an electrokinetic process and bioremediation (EK-bioremediation) enhances the transport of bacteria and nutrients for a more effective biodegradation [46,47]. Adding solubilizing agents, such as surfactants or cyclodextrines, also increases PAH removal [40,47], and in this case, even biosurfactants can be used [39,48]. Ju and Elektorowicz demonstrated that the rhamnolipids coupled with an electrokinetic process increased the solubility into the aqueous phase of phenanthrene contaminating the soil [48].

Consequently, we suggest here to work on contaminated harbor dredged sediments and to use biosurfactant-enhanced PAH solubilization with the aim of coupling it later with a physical-chemical process, such as EK. In natural conditions, *i.e.*, at the aqueous/solid interface, the biosurfactant has to be characterized by a low surface tension to improve PAHs solubility, and this is exactly the case of CLPs. Thus, in a first step, the present study focuses on the feasability of using the anionic CLP, *i.e.*, amphisin, which is produced by *Pseudomonas fluorescens* DSS73, for a PAH solubilization purpose from polluted sediments. Hence, after a PAH’s sorption study on the coarse and finest particles of a model sediment, the efficiency of amphisin will be evaluated on the desorption of a PAH mixture from kaolin. As regards to the *in situ* process, a convenient approach consists in direct addition of amphisin in sediments. An alternative solution would be amendment of the bacteria in the polluted sediments where they will grow and produce their biosurfactant. In that case, unlike classical bioaugmentation, bacterial addition to sediments will not lead to the pollutant biodegradation but solely to the biosurfactant production. Thus, in a second step, the feasability of *P. fluorescens* DSS73 amendment will be evaluated. Therefore, the effect of three model PAHs, taken separately, will be studied on *P. fluorescens* DSS73 physiology, in erlenmeyer flasks and in low oxygen growth conditions. Then to mimic *in situ* conditions, the effect of estuarine water will be also investigated. Thereafter, the biosurfactant production in laboratory conditions or in real estuarine water will be verified.

## 2. Results and Discussion

### 2.1. Purity of Produced Amphisin by *Pseudomonas fluorescens* DSS73

**Figure 1.**
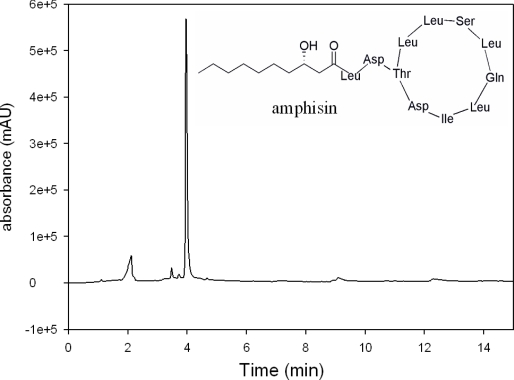
HPLC-UV chromatogram (λ = 210 nm) of the freeze dried biosurfactant produced by *P. fluorescens* DSS73, the amphisin and its structure.

In order to verify the production of biosurfactant by *P. fluorescens* DSS73 grown on DMA, at 8 °C, the surface tension of the rinsing solution was evaluated at 32.1 ± 0.9 mN·m^−1^. Compared to the surface tension of the standard (*i.e.*, 71.7 ± 0.3 mN·m^−1^), this value, which is lower than 40 mN·m^−1^, indicates the presence of a biosurfactant [49]. After purification and lyophilization, the purity of the biosurfactant, described earlier as amphisin [20,50] was analyzed by HPLC-UV. On the chromatogram presented in Figure 1, a single peak appeared, revealing an ultra-majority production of amphisin by *P. fluorescens* DSS73 and not a mixture of different CLPs.

### 2.2. PAHs Adsorption at Water/Sediments Interface and Their Mobilization by Amphisin

#### 2.2.1. PAHs Adsorption on Dredged Sediments Modeled by Sand, Silt and Kaolin

We worked on a model sediment which was reconstituted to mimic the existing sediments from a disposal site managed by a Norman harbor (France). Indeed such a material is able to adsorb particulary high PAHs concentrations due to its high proportion of fine particles (see Experimental section). Thus, we chose to study the behavior of 15 PAHs (among the 16 U.S. EPA priority PAHs generally found in the environment) at various water/solid interfaces, to understand which of the sediment constituents is the more retentive.

**Figure 2.**
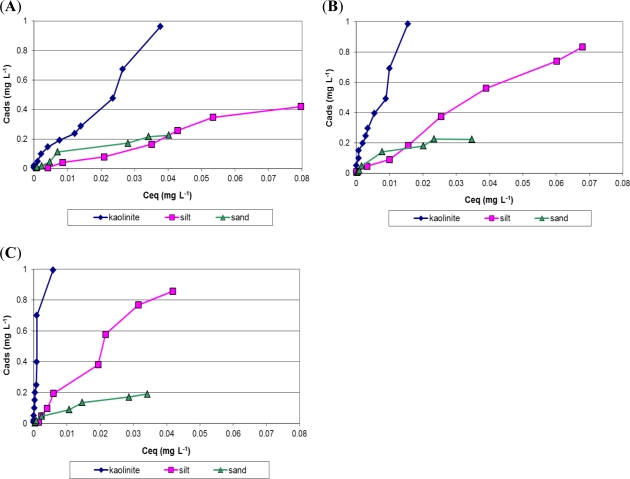
Adsorption isotherms of different PAHs at water/sand, water/silt and water/kaolin interfaces at 25 °C. (**A**) phenanthrene; (**B**) pyrene; (**C**) indeno[*1,2,3,cd*]pyrene.

Only the adsorption of some of the PAHs is plotted in Figure 2 (for more clarity), *i.e.*, phenanthrene, which represents a low weight PAH (Figure 2A), pyrene, which represents an intermediary PAH (Figure 2B) and indeno[*1,2,3,cd*]pyrene, which represents a high weight PAH (Figure 2C). The adsorption isotherms show the interfacial exchanges between water and sand, silt or kaolin, the main components of the harbor sediment. Two main trends are observed through these isotherms. Firstly, PAHs do not adsorb in the same way on the surface of the different sediment constituents. PAH adsorption on sand is markedly weaker than on kaolin, and silt presents an intermediate. Indeed, all PAH adsorption isotherms reach a saturation level for sand at approximately 0.2 mg·L^−1^ (1.8 μg of each PAH per gram of dry sand). This saturated value is higher on the silt (between 0.4 mg·L^−1^ and approximately 0.9 mg·L^−1^ according to tested PAH, *i.e.*, between 3.6 and 8.1 μg·g^−1^). The saturation level is not reached on the kaolin surface in the studied concentration range. These results demonstrated that PAHs concentrate more on the finest particles, where the proportions exceed 9 μg·g^−1^ of dry kaolin. Secondly, PAH adsorption is related to its structure, *i.e*., the number of aromatic ring (three or four ring PAHs, respectively, in Figure 2 (A,B), or six ring PAHs in Figure 2C). Adsorption isotherms from fine kaolin particles are almost parallel to the y-axis for the very heavy PAHs (from benz[*a*]anthracene to indeno[*1,2,3,cd*]pyrene), meaning that they are so strongly adsorbed on the clay that we can really speak about “sequestration” of PAHs by this major constituent of the sediment.

Tests were also carried out on the presence of sodium chloride (10 g·L^−1^), since the water present in the sediment pond, closed to the harbor ecosystem, is briny. The presence of salt had rather little effect on PAHs adsorption on sediments (data not shown): only light PAHs were slightly less adsorbed on fine particles, because they were a little more soluble in the aqueous phase, with a higher ionic strengh.

Finally, PAHs adsorption was performed on the reconstituted sediment matrix (mixture of sand, silt and kaolin). Organic matter (OM) was introduced at 1 or 5% w/w. In such conditions, PAHs (especially the heaviest ones) were more strongly adsorbed, and this phenomenon was inceased when the OM concentration rose (data not shown).

Having shown how the adsorption mechanism works, our goal was now to manage to desorb, *i.e*., to transfer into the aqueous solution, a part of the strongly adsorbed PAHs on sediment particles, with the aim of their removal by an EK-bioremediation process. Indeed, the mobilization of sequestred PAHs by an anionic micellar system could make it possible not only to enhance the PAH solubilization in water, but also to enhance the migration towards the anodic compartment of an electromigration pilot. Our purpose was also to evaluate the desorption phenomenon in conditions close to real ones, observed in sediment disposal sites: the concentration of studied PAHs were lower than those usually found in literature [51,52], and the tested PAHs were chosen to cover different PAH characteristics (hydrophobicity, more or less complex structure).

#### 2.2.2. PAHs Solubility Enhancement with the Help of Amphisin

**Figure 3.**
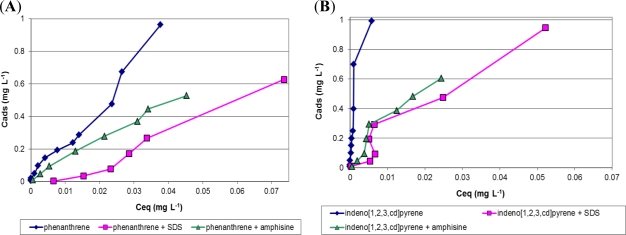
Adsorption isotherms of different PAHs at water/kaolin interface in the presence of SDS (8 × 10^−2^ mol·L^−1^) or amphisin (7.2 × 10^−4^ mol·L^−1^) and in their absence at 25 °C. (**A**) phenanthrene; (**B**) indeno[*1,2,3,cd*]pyrene.

Based on previous results, kaolin seems to be the most powerful PAHs adsorbent through sediment components, so amphisin efficiency was first evaluated on kaolin. The mixture of 15 PAHs was used to pollute our model sediment. In this part of the study, for more clarity, we report only phenanthrene, representing low weight PAHs (Figure 3A), and indeno[*1,2,3,cd*]pyrene, representing heavier PAHs (Figure 3B).

Contaminant solubilization is enhanced by any surfactant, synthetic [39] or biological [53], through micellar solubilization. Hydrophobic PAHs are entrapped inside the micelle, whose core is more hydrophobic than the matrix bulk, and PAHs solubility is greatly enhanced. As reported by Lai [54], the PAHs removal efficiency increased with a biosurfactant concentration increase, but it is not significantly related to the contact time.

First, the added amphisin concentration, *i.e*., 0.1 g·L^−1^ into the system, may be slightly above its CMC, which should be closer to other CLPs, for example 0.054 g·L^−1^ for viscosin [55]. Unfortunately, at this concentration, no clear PAHs mobilization was obtained for any PAHs, light or heavy. In fact, a small amphisin quantity should be adsorbed on the solid/liquid interface, increasing the effective CMC related to this model medium, which is higher than the CMC in water.

After adding more amphisin (1 g·L^−1^, *i.e*., 7.2 × 10^−4^ mol·L^−1^), an important decrease in the slope of the adsorption isotherms was observed as shown in Figure 3. At such a concentration, amphisin kept a greater amount of PAHs in the aqueous phase. This PAH mobilization was observed for light PAHs, such as phenanthrene (Figure 3A), and for the heavier (and more adsorbed) ones, such as indeno[*1,2,3,cd*]pyrene (Figure 3B). At first sight, PAHs solubilizing enhancement from fine kaolin particles was managed with quite a great quantity of amphisin. To compare with a conventional synthetic surfactant, namely sodium dodecylsulfate (SDS), 10^−2^ mol·L^−1^ (2.88 g·L^−1^) of SDS was added to evaluate the efficiency of the solubilizing enhancement. As for amphisin, this chosen SDS quantity was close to its CMC (8 × 10^−3^ mol·L^−1^) and did not significantly allow PAH mobilization in an aqueous phase. Yet, with a concentration “ten-fold” over its CMC (23.07 g·L^−1^), SDS enhanced the PAH’s solubilization in an aqueous phase in equivalent proportions to the amphisin for heavier PAHs (Figure 3B). In summary, amphisin, an anionic CLP, presents great potential to mobilize PAHs in aqueous phase, even the most adsorbed ones on the kaolin surface, and its efficiency is noteworthy with 20-times less (in mass concentration) or 110-times less (in molar concentration) that of the anionic synthetic SDS.

Next we tested if the strain is able to grow and still to produce amphisin in the presence of pollutants and in the real interstitial aqueous medium of the dredged sediments.

### 2.3. Effect of Three Model PAHs on *Pseudomonas fluorescens* DSS73 Growth Kinetics

#### 2.3.1. Growth in Erlenmeyer Flasks

Pyrene, fluoranthene or phenanthrene was introduced, as model PAHs, before inoculation of Davis Minimal Broth (DMB) by *P. fluorescens* DSS73 to follow its growth in erlenmeyer flasks.

As PAH concentrations in the dredged sediments are generally under their solubility limit, two concentrations were tested: one just below the solubility limit and one slightly above.

**Figure 4.**
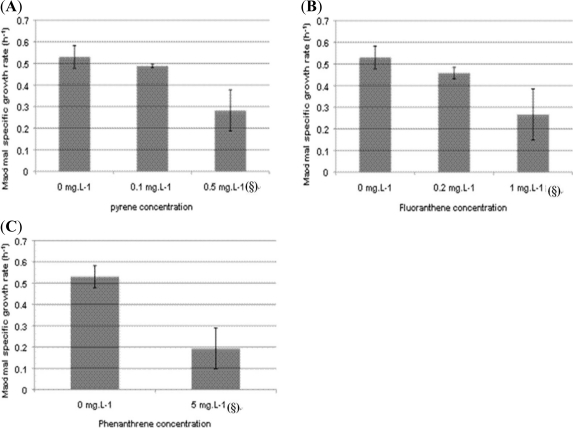
Effect of PAH concentration on maximal specific growth rate of *P. fluorescens* DSS73 in DMB. (§: PAH concentration higher than its solubility limit). (**A**) pyrene; (**B**) fluoranthene; (**C**) phenanthrene. All experiments were done in triplicate.

As shown in Figure 4, for all the tested PAHs concentrations, *P. fluorescens* DSS73 was able to grow. At concentrations lower than PAHs saturation, the maximum specific growth rates, μ_max_, were in the same range with or without PAH, thus the presence of pollutants did not significantly modify bacterial development. However, when the growth medium was saturated by PAHs, the bacterial growth was more disturbed: μ_max_ decreased drastically for each of the three PAHs.

Culturing in erlenmeyer flasks favors bacterial growth in regard to those existing growth conditions in dredged sediment disposal sites, where the oxygenation of the matrix is weaker. So growth tests were also performed in oxygen-limited conditions.

#### 2.3.2. Growth in Oxygen-Limited Conditions

To better mimic *in situ* parameters, a similar study was made with a microplate reader. With this apparatus, same growth rates were obtained with regard to erlenmeyer cultures, when 100 μL of culture were used in 400 μL microplate wells (data not shown). Cultures performed with 200 μL exhibited reduced growth that can be attributed to a lack of oxygen. Thus, the maximal growth rate was 0.53 h^−1^ for growth in erlenmeyer flasks *versus* 0.33 h^−1^ for growth in the microplate.

**Figure 5.**
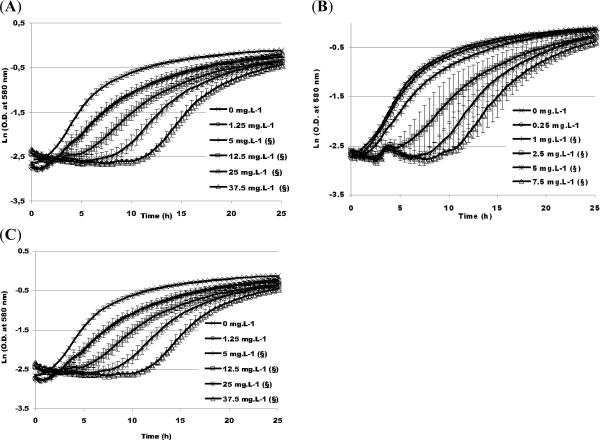
Effect of different PAH concentrations on the growth kinetics of *P. fluorescens* DSS73 in DMB in oxygen-limited conditions. (§: PAH concentration higher than its solubility limit). (**A**) pyrene; (**B**) fluoranthene; (**C**) phenanthrene. All experiments were done in triplicate.

**Figure 6.**
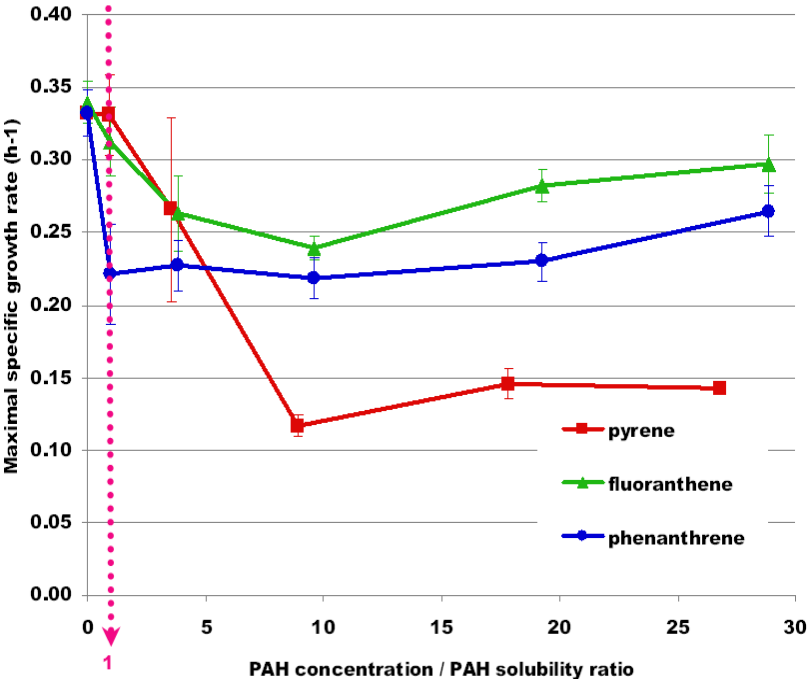
Influence of the concentration/solubility ratio of each PAH on the maximal specific growth rate (μ_max_) of *P. fluorescens* DSS73 in DMB in oxygen limited conditions. All experiments were done in triplicate.

As represented in Figure 5, *P. fluorescens* DSS73 growth was stunted with increasing PAH concentration. The higher the PAH concentration was, the longer the lag phase. For some growth kinetics, an important experimental error was observed especially at high PAH concentrations. This was related to the high variability of the lag phase observed on the three replicates. In Figure 6, the previous results were summarized and normalized: μ_max_ was reported *versus* PAH concentration normalized by PAH solubility. It appeared that when the PAH concentration was lower than its solubility (e.g., concentration over solubility ratio lower than 1), μ_max_ was not significantly affected by PAHs (*p* = 0.977 and *p* = 0.305 for pyrene and fluoranthene, respectively, with a *t*-test) except for phenanthrene (*p* = 0.007). When the PAH concentration was higher than the solubility limit, the maximal specific growth rate was drastically reduced with regard to a medium without contaminant. Similar trends were observed in both conditions with or without oxygen limitation.

Pyrene presented a greater inhibitory effect on *P. fluorescens* DSS73 growth than the two other PAHs. Its structure is made of four juxtaposed aromatic cycles, whereas three cycles exist for the two others. Increasing the number of aromatic cycles may lead to a greater negative impact on *P. fluorescens* DSS73 growth.

To conclude, *P. fluorescens* DSS73 is able to grow in the presence of PAHs in laboratory conditions.

#### 2.3.3. Growth Experiments in Estuarine Water

**Figure 7.**
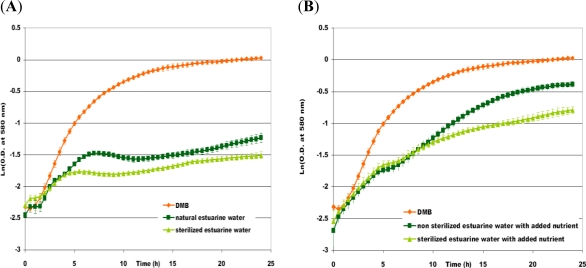
Growth kinetics of *P. fluorescens* DSS73 (**A**) in sterilized or natural estuarine water; (**B**) in sterilized or unsterilized water supplemented with nutrients, compared to DMB.

To approach a more *in situ* condition, the growth study in limited oxygenation was repeated, this time, with estuarine water feeding the dredged material disposal site of a Norman harbor as growth medium. Several growth experiments were performed with sterilized or crude estuarine water, with or without nutrient addition. In Figure 7, the growth kinetics were plotted in the case of estuarine water without added nutrients (Figure 7A) and supplemented with nutrients (Figure 7B). The addition of nutrients modified the profile of the growth curve. Indeed, *P. fluorescens* DSS73 growth curves, in Figure 7B, showed two phases with two different μ_max_ (about 0.2 h^−1^, then 0.1 h^−1^) separated by a lag phase of 2 h. An explanation of this diauxy phenomenon may be that *P. fluorescens* DSS73 first metabolized the added nutrients, which acted as a growth stimulant, and then used the estuarine water as the second source nutrients.

With or without sterilization, growth curves showed the same trends during exponential phase, with similar growth rate values. Then, the sterilization did not improve growth, as would be expected. Indeed, it seems that the indigenous flora of estuarine water did not inhibit *P. fluorescens* DSS73 growth. Final maximum optical density (OD_max_) at the end of the stationary phase was higher in crude estuarine water than in sterilized water, in the case of nutrient addition. An explanation could be that *P. fluorescens* DSS73 may inhibit other microorganisms and even metabolize their residue. Moreover, the OD_max_ values reached in crude estuarine water were equal to 0.4 without addition of nutrient and 0.7 for the supplemented growth, which leads to the conclusion that adding nutrient improves biomass production.

To conclude, *P. fluorescens* DSS73 is able to grow in estuarine water and addition of nutrients favors the growth.

### 2.4. Evaluation of Amphisin Production

#### 2.4.1. Amphisin Production in the Presence of PAHs

After demonstrating the potential for *P. fluorescens* DSS73 to grow in the presence of PAHs, we could also show that the strain is still able to produce biosurfactants. Table 1 compiled the surface tension measured before inoculation and after bacterial growth in erlenmeyer flasks for each of the three PAHs, in higher concentration than their solubility limit. For pyrene and fluoranthene, the surface tension decreased slightly, but for phenanthrene no modification was noted. The difference of surface tension did not reach 20 mN·m^−1^ [52] after bacterial growth, which could suggest that no biosurfactant was produced. However, it was interesting to note the enormous relative standard deviation associated to the mean values of surface tension after growth (between 9.4% and 18.8%), so interfacial exchanges were effectively modified. It may be correlated to the PAH’s entrapment into amphisin micelles; amphisin mobilized at interface DMB/PAH could not help to decrease the surface tension relative to DMB/air interface. Although it was not clearly established, amphisin could be produced in PAH’s presence.

#### 2.4.2. Amphisin Production by *Pseudomonas fluorescens* DSS73 Growing in Estuarine Water

As already shown, *P. fluorescens* DSS73 growth may occur in estuarine water, and it was still able to produce amphisin (Table 2).

In all cases, after bacterial growth in estuarine water, the surface tension was less than 40 mN·m^−1^, criterion required to detect biosurfactant production according to Carillo [49]; moreover the growth in estuarine water resulted in a decrease of surface tension of more than 30 mN·m^−1^. Without any doubt, amphisin is produced by DSS73 in estuarine water, with or without nutrient addition.

## 3. Experimental Section

### 3.1. Microorganism

*P. fluorescens* DSS73 is a strain from sugar beet rhizospheric environment and was graciously provided by Dr. O. Nybroe (Royal Veterinary & Agricultural University-Thorvaldsensvej, Denmark).

### 3.2. Growth Medium

The bacteria were grown in Davis Minimal Broth (DMB: 30 mmol·L^−1^ K_2_HPO_4_, 14 mmol·L^−1^ KHPO_4_, 0.4 mmol·L^−1^ MgSO_4_, 7.6 mmol·L^−1^ (NH_4_)_2_SO_4_, 120 mmol·L^−1^ glucose, and 1 mL of trace element solution per liter (pH 7.3). The trace element solution contained per liter of pure water 20 mg of CoCl_2_·6H_2_O, 30 mg of H_3_BO_3_, 10 mg of ZnSO_4_·7H_2_O, 1 mg of CuCl_2_·2H_2_O, 2 mg of NiCl_2_·6H_2_O, 3 mg of NaMoO_4_·2H_2_O, 10 mg of FeSO_4_·7H_2_O, and 2.6 mg of MnSO_4_·H_2_O. The glucose and trace elements were sterilized separately by filtration (Steriltop, Millipore, France) and aseptically added to the rest of the medium. Growth on solid medium was performed on DMA (Davis Minimal Agar) with the same composition as DMB supplemented with agar (15 g·L^−1^).

Cultures were also carried out in estuarine water that was collected in sterile bottles, in an estuarine canal feeding the dredged material disposal site (Normandy, France). Then the estuarine water was stored at 4 °C and used for the cultures in the following 24 h. The pH of the water was adjusted to 7. Bacterial growth was tested in unsterilized or sterilized (by filtration) estuarine water. Cultures were done using unsterilized or sterilized estuarine water as aqueous base with DMB components. The addition of nutrients resulted in ion precipitation. The precipitate was eliminated by centrifugation (7000 g, 10 min).

### 3.3. Biosurfactant Recovery

To test for biosurfactant production, the bacterial colonies were grown on Davis minimal agar. DMA culture plates were inoculated with 40 μL of stocked suspension. After incubation for at least a week at 8 °C, bacterial colonies were scraped off, transferred to 15 mL of sterile mineral water and dispersed by agitation. This mineral spring water was used as its low level in mineral charge ensures a good repeatability and high values for tensiometry. After centrifugation (30 min, 4 °C, 18,000 g), the surface tension of the supernatant could be determined by the direct method of the pendant drop using a G40 goniometer (Krüss, France).

The biosurfactant purification was performed by liquid-liquid extraction: 100 mL of aqueous supernatant completed with 4 × 10^−3^ mol·L^−1^ of trifluoroacetic acid (TFA), was mixed and agitated one hour with 150 mL of ethyl acetate, and then transferred into a separatory funnel. A second extraction of the aqueous phase was carried out each time. After collection of all organic phases, they were evaporated at 40 °C and 250 mbar with the rotary evaporator, to obtain approximately 10 mL of extract. In order to eliminate free water, this extract was then freeze-dried (semi-pilot freeze-drier SMH15, Usifroid, Maurepas, France), the sublimation took at least 2 days. The solid lyophilized sample was then solubilized in acetonitrile and was analyzed in liquid chromatography (HPLC) with UV detection (at 210 nm): The purified amphisin was separated on a hydrophobic C_18_ bonded column (Beckman, Fullerton, USA) (4.6 mm × 250 mm, particles of 5 μm), using as a mobile phase a solvent mixture composed of: 25% water containing 0.1% TFA and 75% acetonitrile, at 1 mL·min^−1^.

### 3.4. Model Sediment for PAH Adsorption

A sediment is first characterized through its granulometry, its mineral composition, its water and organic matter (OM) content. The granulometric classification, used for sediments in geology (French standard NF P18-560), differentiates largest blocks, rollers, stones or gravels, of diameters greater than 2 mm, sands (diameter ranging between 20 μm and 2 mm), silts (2 to 20 μm) and clay sludges or muds (diameters less than 2 μm). The reconstituted studied sediment contained 5% sand, with a particle diameter between 125 and 315 μm (Sika, Hostun, France), 75% silt from the Normandy shelves, with a diameter between 4 and 100 μm (CETE, Rouen, France) and 20% kaolinite clay, with a diameter being less than 80 μm, including 54% of particles less than 2 μm (Imerys, Poigny, France).

### 3.5. PAH Quantification at the Water/Sediment Interface

The PAH adsorption at the water/sediment interface was carried out in reactors containing 10 g of model sediment for 90 mL of aqueous phase (ratio 10/90 in weight or 111 g of dry sediment per liter), maintained at a constant temperature (25 °C) in a thermostated bath (Fisher Bioblock Scientific, IIlkirsh, France) and under agitation (300 rpm). In many experiments, sodium chloride could be added into water at a concentration of 10 g L^−1^. In other experiments, organic matter could be mixed with the model sediment, in variable proportions (1 to 5% w/w). This organic matter was obtained from the decomposition of a vegetable material (VEOLIA-France): it was then dried 48 h at 50 °C, crushed and sieved to obtain particle diameters less than 355 μm. PAH introduction in reactors was done from a known concentration (*C*_o_) of a stock solution of 16 PAHs (100 mg·L^−1^) in water. When thermodynamic equilibrium was reached (2 h for kaolin alone, 12 h for sand alone, 4 h for the model sediment), the solid phase was eliminated by centrifugation (40 min, 11,200 rpm). Then an enrichment protocol by SPE (solid phase extraction) was done to concentrate the traces of non-adsorbed PAH still present in the aqueous phase at the end of the equilibrium step (*C*_eq_). Indeed, direct PAH analysis and quantification by HPLC would have been impossible without SPE enrichment because non-adsorbed PAHs were found under the limits of detection of the fluorimetric detector. It must be added that even after centrifugation, ultra-fine clay particles remained in suspension in liquid phase; so SPE cartridges Strata X (Phenomenex), containing 60 mg of polymeric solid phase, had to be coupled with Phenex 0.45 μm filters, made of inert Teflon (Phenomenex), to eliminate colloid particles. Moreover, to improve PAH extraction yields, which had to be higher than 80%, it was necessary to homogenize the aqueous supernatant by adding 20% acetone in volume (HPLC grade, Fisher Scientific) before starting its percolation through the SPE cartridge. This step also made it possible to avoid the PAH sorption on the flask’s glass walls, leading to high PAH losses, a particularly critical phenomenon when traces of PAH have to be analyzed quantitatively. SPE cartridges were first conditioned with methylene chloride, methanol and a mixture of acetone/water (20/80 v/v) (all solvents were of analytical grade, from Fisher Scientific France). Then, after percolation of the aqueous supernatant through the SPE cartridge associated to a filter, extracts were eluted with 5 mL of methylene chloride (HPLC grade, Fisher Scientific).

Methylene chloride was evaporated under nitrogen flow (after adding 60 μL of dimethylsulfoxide) for solvent change; then the sample was solubilized again in 4 mL of acetonitrile (HPLC grade, Fisher Scientific). 10 μL of this sample was injected on a PP Envirosep C_18_ bonded column (Phenomenex; 150 mm × 4.6 mm, particles of 5 μm), with a flow rate of 1 mL·min^−1^ controlled by two Beckman Coulter Gold 126 pumps.

Table 3 shows the solvent gradient conditions during chromatographic elution as well as the programmed excitation and emission wavelengths of the fluorimetric detector Prostar 363 (Varian, Palo Alto, USA). After HPLC analysis, each of the 16 PAHs could be individually quantified. Their aqueous concentration could be evaluated (as *C*_eq_ at equilibrium in water) and consequently, knowing their initial introduced concentration (*C*_o_), C_ads_ could be calculated by a simple subtraction as the concentration adsorbed on sediment. Adsorption isotherms at 25 °C were then established in varying *C*_o_ and in plotting *C*_ads_ = *f*(*C*_eq_).

### 3.6. Solubility Enhancement of PAHs by Amphisin and SDS

The mixture of 16 PAHs was introduced into reactors containing kaolin, as further described. After mixing them for one hour at 25 °C, the purified and lyophilized biosurfactant was then introduced into the system at two different concentrations: 0.1 g·L^−1^ and 1 g·L^−1^. After three hours of equilibrium, the mixture was centrifuged and the non-adsorbed PAH were quantified as described elsewhere. Other similar experiments were done with a synthetic surfactant, the sodium dodecyl sulfate SDS (provided by Sigma-Aldrich), introduced in a similar way at 2.88 g·L^−1^ or 23.07 g·L^−1^ in the aqueous phase.

### 3.7. Bacterial Growth Conditions

#### 3.7.1. Growth in Erlenmeyer Flasks with PAHs

Seed culture was carried out in 10 mL of DMB overnight at 28 °C on a rotary shaker (180 rpm). In a 500 mL erlenmeyer flask, 50 mL of DMB supplemented with PAH from stock solution in acetone, was inoculated with an aliquot of seed culture in order to obtain an initial OD of 0.05. The tested PAH concentrations were 0.1 and 0.5 mg·L^−1^ for pyrene, 0.2 and 1 mg·L^−1^ for fluoranthene and 5 mg·L^−1^ for phenanthrene. The culture conditions were identical as for the seed culture. Bacterial density was determined by measuring optical density at 580 nm (Spectronic 601 spectrophotometer). For each PAH concentration, triplicate flasks were employed.

#### 3.7.2. Growth in Oxygen-Limited Conditions

Cultures were made in polystyrene microtitration plates. 180 μL of DMB was deposited in each 400 μL well and inoculated by 20 μL of seed culture in DMB incubated at 28 °C. To study the effect of PAH on bacterial growth, PAH was added to the mixture in the wells, from PAH stock solutions in acetone, to obtain final concentrations of 0.125, 0.5, 1.25, 2.5, 3.75 mg·L^−1^ for pyrene, of 0.25, 1, 2.5, 5, 7.5 mg·L^−1^ for fluoranthene, and of 1.25, 5, 12.5, 25, 37.5 mg·L^−1^ for phenanthrene. To evaluate the influence of estuarine water on growth kinetics, DMB was replaced by estuarine water.

For each condition, *P. fluorescens* DSS73 growth was followed continuously in triplicate in a thermoregulated spectrometer (Xenius, Safas, Monaco) at 28 °C under rotary revolution (180 rpm).

### 3.8. Tensiometry

The surface tension was tested on culture supernatants after centrifugation (30 min, 4 °C, 18,000 g) from 50 mL of liquid cultures. The home-made surface tensiometer worked on the principle of the Wilhelmy plate method. All surface tensions were measured in triplicate. The measurement validity was standardized with mineral spring water of weak mineral charge (71.7 ± 0.3 mN·m^−1^).

## 4. Conclusions

Biosurfactant produced by *Pseudomonas fluorescens* DSS73 seems very promising with regard to mobilization in an aqueous phase of strongly adsorbed PAHs on the finest sediment particles. It was almost as efficient as conventional synthetic surfactants, such as SDS, for solubilizing the heaviest and most strongly adsorbed PAHs. However, mobilization by amphisin needs much lower quantities than those required with the synthetic surfactant. Future research needs to investigate if amphisin is also efficient in solubilizing PAHs even when another retentive material, *i.e.*, organic matter, enters the composition of the sediment; afterwards, we will also have to prove that the ionic amphisin, after its increased solubilizing effect, will be able to enhance PAH mobility through the sediment via an electromigration process. Indeed, EK remediation is a promising *in situ* method for grounds or sediments of low permeability, and is adapted to numerous pollutant types.

This study also proved that the strain was able to produce the cyclolipopeptide in hostile growth conditions corresponding to real environment, *i.e*., in estuarine water feeding a dredged material disposal site. *P. fluorescens* DSS73 was also able to produce amphisin, in the presence of a relatively high PAH contamination, and even with low oxygen growth conditions. These very promising results suggest that bioaugmentation of the biosurfactant producer, *P. fluorescens* DSS73, could be conceivable *in situ*. As amphisin is biodegradable (which is an advantage for the environment), single amendement is not sufficient when long periods are necessary to perform pollution attenuation, which is certainly the case when EK remediation is performed *in situ*, on a large scale. Moreover, EK-bioremediation of PAHs via anionic micelles needs certainly a continuous addition of the biosurfactant, because aggregates migrate towards the anodic compartment and are consequently impoverished inside the sediment during the treatment. Thus, it would be a real advantage to continue the mobilization and migration process with biosurfactant production *in situ*.

## Figures and Tables

**Table 1.** Surface tensions and associated standard deviations of *P. fluorescens* DSS73’s growth in the presence of PAHs, above their solubility limit. All experiments were performed in triplicate.

**Tested PAH**		**Surface Tension (mN·m^−1^)**	

**Name**	**Concentration (mg·L^−1^)**	**Before Growth**	**After Growth**
Pyrene	0.5	52.2 ± 0.3	48.5 ± 9.1
Fluoranthene	1.0	55.0 ± 0.1	43.9 ± 7.1
Phenanthrene	5.0	55.9 ± 0.1	55.6 ± 5.2

**Table 2.** Surface tension and associated standard deviations of *P. fluorescens* DSS73’s growth in estuarine water with or without nutrients. All experiments were performed in triplicate.

**Tested Medium**	**Surface Tension (mN·m ^1^)**		**Amphisin Production**
	**Before Growth**	**After Growth**	
DMB	69.3 ± 0.9	30.4 ± 0.5	+
Estuarine water without nutrient	60.9 ± 0.5	32.8 ± 0.2	+
Estuarine water with nutrients		29.8 ± 0.6	+

**Table 3.** HPLC elution gradient for the analysis of the 15 priority PAHs (defined by U.S. EPA) plus benzo[*e*]pyrene, and conditions for the fluorimetric detection.

**Time (min)**	**Elution Gradient**	**λ_ex_ (nm)**	**λ_em_ (nm)**	**Detected PAHs**
0	Acetonitrile/water 55/45%	220	330	Naphthalene (1)
5	Acetonitrile/water 55/45%			
7	Linear gradient for 20 min	220	315	Acenaphthene (2)
				Fluorene (3)
10		250	370	Phenanthrene (4)
				Anthracene (5)
13.4		235	420	Fluoranthene (6)
				Pyrene (7)
17		267	385	Benz[*a*]anthracene (8)
				Pyrene (9)
21.7		260	420	Benzo[*e*]pyrene (10)
25	Acetonitrile/water 100/0%			Benzo[*b*]fluoranthene (11)
				Benzo[*k*]fluoranthene (12)
				Benzo[*a*]pyrene (13)
27.4		290	410	Dibenzo[*a,h*]anthracene (14)
				Benzo[*g,h,i*]perylene (15)
29.8		245	500	Indeno[*1,2,3-cd*]pyrene (16)
35	Acetonitrile/water 55/45%	220	330	
